# Stress distribution analysis in anterior teeth caused by several retraction mechanics

**DOI:** 10.1590/2177-6709.26.5.e212042.oar

**Published:** 2021-08-15

**Authors:** Rafael Golghetto DOMINGOS, Almir LIMA, Dalva Cruz LAGANÁ, José RINO, Jorge ABRÃO, João Batista de PAIVA

**Affiliations:** 1 Universidade de São Paulo, Faculdade de Odontologia, Departamento de Ortodontia (São Paulo/SP, Brazil).; 2 Universidade de São Paulo, Faculdade de Odontologia, Departamento de Prótese (São Paulo/SP, Brazil).

**Keywords:** Orthodontics, corrective, Tooth movement techniques, Orthodontic space closure

## Abstract

**Introduction:**

Orthodontic retraction of the anterior teeth is indicated when the patient has a malocclusion with protrusion of the incisors. Several mechanics are indicated to perform this retraction.

**Objective:**

The objective of this study was to compare the strains generated by four different types of retraction mechanics along the roots of the anterior teeth.

**Methods:**

A photoelastic model simulating an arch with first premolars extraction was made. Sixty retraction archwires were prepared, including fifteen for each type of mechanics evaluated: sliding, teardrop loop spring, T-loop spring and double key loop archwire. The strains were observed in two perspectives: occlusal and oblique. In the occlusal perspective, strains were compared among the six anterior teeth. From the oblique perspective, strains were compared among the thirds of the left canine root.

**Results:**

In the occlusal perspective, the teardrop loop spring mechanics presented greater strains, followed by T-loop spring, double key loop archwire and sliding mechanics. In all mechanics, strains were more concentrated in the canines than in the incisors. From the oblique perspective, the teardrop loop mechanics generated greater strains in the cervical regions of the canine, and in the apical regions, no differences were found in strains among the four types of mechanics. In the same mechanics, greater strains were present in the cervical zones.

**Conclusion:**

The teardrop loop spring retraction mechanic presented the greatest mean strain, and the sliding retraction mechanic presented the lowest mean strain on the root of anteroinferior teeth in the occlusal and oblique perspectives.

## RESUMO

**Introdução:**

A retração ortodôntica dos dentes anteriores é indicada quando o paciente apresenta má oclusão com vestibularização dos incisivos. Diferentes mecânicas são indicadas para realizar essa retração.

**Objetivo:**

O objetivo do presente estudo foi comparar as tensões geradas por quatro diferentes tipos de mecânicas de retração nas raízes dos dentes anteriores.

**Métodos:**

Um modelo fotoelástico foi confeccionado simulando uma arcada com os primeiros pré-molares extraídos. Foram preparados 60 arcos de retração, sendo quinze para cada tipo de mecânica avaliada: deslizamento, alça de Bull, alça em T e arco de dupla chave. As tensões foram observadas em duas perspectivas: oclusal e oblíqua. Na vista oclusal, as tensões foram comparadas entre os seis dentes anteriores. Na vista oblíqua, as tensões foram comparadas entre os terços radiculares do canino esquerdo.

**Resultados:**

Na vista oclusal, a mecânica com alça de Bull apresentou maiores tensões, seguida da alça em T, arco de dupla chave e mecânica de deslizamento. Em todas as mecânicas, as tensões se concentraram mais nos caninos do que nos incisivos. Na vista oblíqua, a mecânica com alça de Bull gerou maiores tensões nas regiões cervicais dos caninos; nas regiões apicais, não houve diferenças nas tensões entre os quatro tipos de mecânicas. Dentro de uma mesma mecânica, as maiores tensões estiveram presentes nas regiões cervicais.

**Conclusão:**

A mecânica de retração com alça de Bull apresentou a maior média de tensões, e a mecânica de retração por deslizamento apresentou a menor média de tensões na raiz dos dentes anteroinferiores, nas vistas oclusal e oblíqua.

## INTRODUCTION

Patients with excessive incisors buccal inclination frequently present abnormalities in masticatory function, such as lack of anterior guidance and unsatisfactory facial aesthetics due to lips protrusion and unaesthetic teeth exposure at rest.^1^ ^-^ ^5^ Intending to correct these features, orthodontic treatment plans have been designed to reduce incisor protrusion. In this context, first premolar extractions are often proposed, followed by retraction of the anterior teeth.^1^ ^,^ ^3^

The orthodontist is responsible for managing the particularities of the existing retraction techniques for space closure after extractions. The ideal approach is identified during treatment planning, aiming at achieving an effective dental movement.^5^ Among the existing techniques to retract the anterior teeth, the following techniques can be cited: sliding mechanics,^6^ ^-^ ^8^ teardrop loop spring,^9^ ^-^ ^12^ T-loop spring^5^ ^,^ ^13^ ^-^ ^15^ and double key loop archwire.^16^ ^-^ ^18^

Photoelasticity is one of the available methods for stress analysis over teeth roots. It belongs to a group of convenient techniques for studying the effects caused by orthodontic retraction of anterior teeth, and it is based on birefringence, which is an optomechanical characteristic of transparent polymers.^19^ Numerous studies in orthodontics have used this laboratory method to analyze the strain fields created on the structures of interest after a mechanical load.^17^ ^,^ ^19^ ^-^ ^26^

Despite the frequent evaluation of retraction mechanics by several studies,^1^ ^,^ ^2^ ^,^ ^5^ ^-^ ^18^ it is difficult to find articles that have compared the effects of the stress produced in periodontal tissues on anterior teeth with several space closing techniques. Therefore, this investigation aimed at analyzing and comparing the stress distributions in the following four anterior retraction techniques: sliding mechanics, teardrop loop spring, T-loop spring and double key loop archwire.

## MATERIAL AND METHODS

The tests were performed with a photoelastic model simulating a dental arch in the initial orthodontic anterior retraction phase with first premolars extraction. Roth 0.022-in fixed orthodontic appliance (Abzil, São José do Rio Preto, Brazil) was installed on artificial teeth (Orto-Art; Piracicaba, Brazil) and a dental simulator (typodont) was created with the teeth correctly positioned along a 0.021 x 0.025-in steel archwire (Morelli, Sorocaba, Brazil) without the first premolars. Next, an impression of the typodont was made with silicone (Redelease, São Paulo, Brazil). After silicone polymerization, the wax was removed and a flexible epoxy resin was poured into the impression (Epoxi Glass; Diadema, Brazil). The 0.021 x 0.025-in steel archwire used during the construction of the model was removed, and an orthodontic archwire diagram was made with it as reference for the retraction archwires used on the subsequent mechanical tests. A transparent acrylic resin base fitted to the photoelastic model was used to simulate an absolute anchorage in the posterior segment, intending to concentrate all the resulting retraction forces in the anterior area of the arch.^27^ A mini-implant was placed on each side in the distal area of the base and attached to the mandibular first molar tube with a metallic ligature.

**Figure 1:**
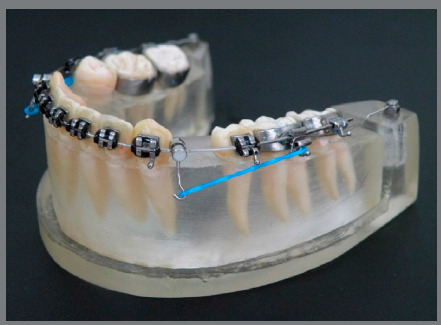
Sliding mechanics test specimen.

**Figure 2:**
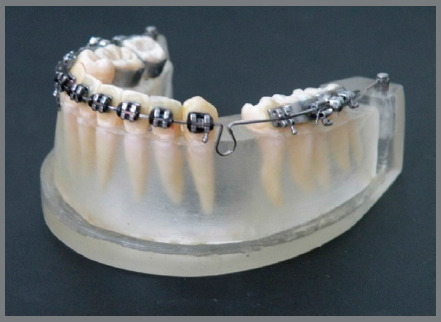
Teardrop loop spring test specimen.

**Figure 3:**
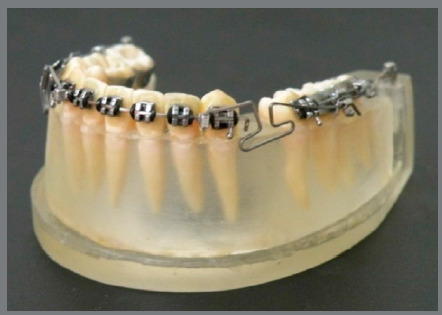
“T” loop spring test specimen.

**Figure 4:**
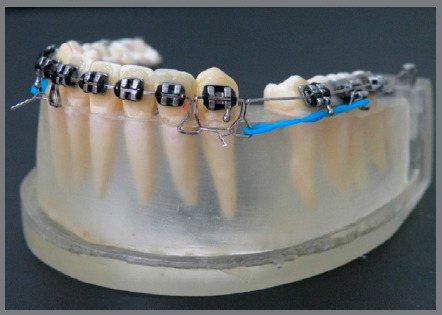
DKL archwire test specimen.

**Figure 5:**
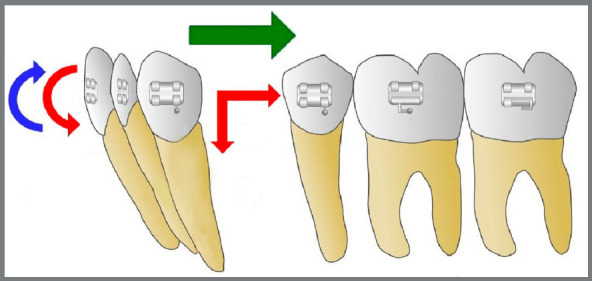
Diagram of the intended moment of force. The vectors in red refer to the force applied on the anterior teeth, generating an intrusive rotation moment that canceled out the extrusive rotation moment, generated by the resistance to the retraction movement (shown in blue). The horizontal vector (in green) is the intended final resultant force.

In Group 1 (sliding mechanics), 0.019 x 0.025-in steel archwires were used. Long hooks were installed bilaterally on the wire in the canine region.^6^ ^-^ ^8^ An elastic power chain was loaded bilaterally from the first molar tube to the hook, to make the retraction force (Fig 1). In Group 2, 0.019 x 0.025-in steel retraction archwires were used with bilateral teardrop loop springs positioned 2 mm distal from the canine brace. A Gable bend of 15 degrees was made^9^ ^-^ ^12^ (Fig 2), and the retraction force was made by loop activation. In Group 3, a 0.021 x 0.025-in steel arch was sectioned in three parts, which were installed in the two posterior segments and in the anterior segment. A cross tube was welded to the wire between the canine and the lateral incisor. Bilaterally, the activation was performed with a T-loop spring made of 0.017 x 0.025-in titanium molybdenum alloy (TMA) wire that was attached to the anterior section cross tube and loaded onto the auxiliary slot of the first molar tube (Fig 3). This loop was displaced to the anterior portion of the interbrackets space from the canine to the second premolar with a 45-degree Gable bend close to the molar tube.^5^ ^,^ ^13^ ^-^ ^15^ In Group 4, prefabricated 0.019 x 0.025-in steel retraction archwires with double key loops (DKL) positioned 1 mm mesial to and 1 mm distal from the canine brackets were used. Bilaterally, these loops were tied by metallic ligatures until the segment between the mesial loops reached 0.5 mm of deflection. The retraction force was applied by an elastic power chain loaded from the distal canine loop to the hook of the first molar tube^16^ ^-^ ^18^ (Fig 4). A total of sixty retraction archwires were prepared (fifteen for each group). Figure 5 shows a diagram that illustrates the moment of force expected for the applied mechanics.

With a circular polariscope, the absence of residual stresses was verified in the photoelastic model prior to the beginning of each test, and the isochromatic photoelastic fringes that appeared after the activation of each retraction mechanic were observed. To prevent bias regarding an eventual fatigue of the photoelastic model, the activations were performed by alternating the groups always in the same sequence - 1, 2, 3, 4, 1, 2, 3, 4, and so on, until the fifteenth test of each group was carried out. In the end, the photoelastic model still proved viable if more activations were needed. The activation force was standardized at 240 g per side, which produced a total of 480 g force in the activation moment of the retraction mechanics. After each activation, standardized photographs were taken with the aid of marks made in the photoelastic model fixation base and in the photographic camera tripod.

These photographs were taken in two predefined perspectives according to the evaluated view of this study. In an occlusal perspective, the model was positioned uprighted, with the camera lens frame 90º in relation to the occlusal plane. In the oblique perspective, the model was positioned so that the camera lens was positioned 90º from the left canine’s vestibular face, with the canine centered in the image. The collected images were transferred to a computer for visual analysis. Six zones were evaluated in the occlusal perspective: the lingual zones of the four incisors and distal zones of the two canines. Five zones of the left canine root were evaluated in the oblique perspective: cervical-mesial (CM), apical-mesial (AM), apical (A), distal-apical (DA) and cervical-distal (CD). In the qualitative analysis, the photoelastic fringes were described according to their density and morphology - high strained zones are indicated by small space and fine fringes.^19^ In the quantitative analysis, the photoelastic fringes expressed in each evaluated zone were classified with an ordinal number, as described by the American Society for Testing and Materials (ASTM - D4093). The absence of strain is commonly depicted by large black or gray areas. The different coloration among the lines shows the transition from one to another fringe. The first one, black colored, is the one of order zero; the one of violet color, order one; the violet/blue transition, order two; transition from red to green, order three. From the fringe of order three, the subsequent fringes are always counted in the transition from red to green.^19^

## STATISTICAL ANALYSIS

The photoelastic fringe data were analyzed twice by the intraclass correlation coefficient (ICC) with 95% confidence intervals, to verify the agreement/reproducibility between the first and second measurements, and the measurement repeatability was calculated.^28^ The values for the first evaluation were subjected to the Kolmogorov-Smirnov test and classified as nonparametric, so the Friedman test^29^ was chosen to compare the outliers of the four types of mechanics. If statistical significance was shown by this test, multiple nonparametric paired comparisons were performed to verify the types of mechanical differences in the fringes. All tests were performed with a significance level of 5%.

## RESULTS

**Figure 6:**
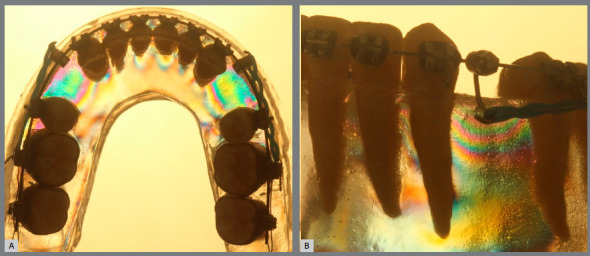
Group1 photoelastic image: A) occlusal perspective; B) oblique perspective.

Qualitative analysis of the four groups in this study allowed the observation of the following characteristics:

» Group 1 (sliding mechanics): In the occlusal perspective, few photoelastic fringes appeared on the canine distal zones, extending until the second premolar mesial area. In the incisor lingual zones, less intense fringes were observed when compared to canine distal zones. In the oblique perspective, mild color alterations were observed in the three apical zones. Nevertheless, a more evident fringe sequence of colors was observed in the cervical zones, principally in the cervical-distal zone (Fig 6).

**Figure 7:**
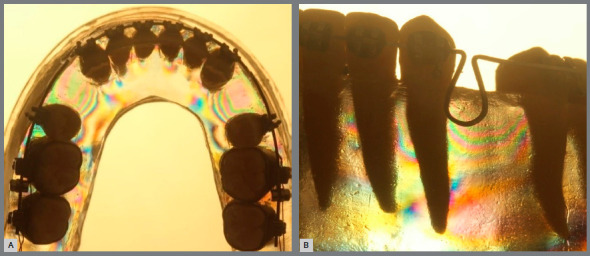
Group 2 photoelastic image: A) occlusal perspective; B) oblique perspective.

» Group 2 (teardrop loop spring): In the occlusal perspective, more intense colors and a greater sequence of fringes were observed bilaterally in the canine distal zones. In the incisor lingual zones, less intense fringes appeared when compared to the canine distal zones. In the oblique perspective, a more perceptible color alteration was observed in the apical zones. In the cervical zones, an evident increase in the fringe sequence was observed (Fig 7).

**Figure 8:**
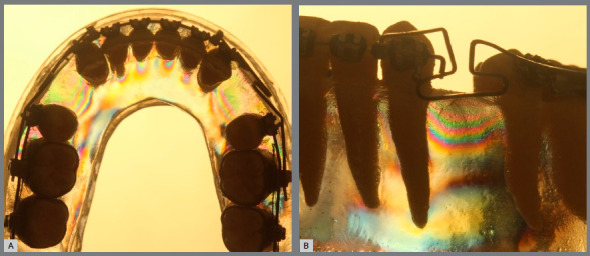
Group 3 photoelastic image: A) occlusal perspective; B) oblique perspective.

» Group 3 (T-loop spring): In the occlusal perspective, the color alterations and fringe sequences were similar to Group 2 in intensity and quantity. In the oblique perspective, a less intense color alteration was observed in all the apical zones. In the cervical zones, the distal segment presented a more evident fringe sequence when compared to the mesial zone (Fig 8).

**Figure 9:**
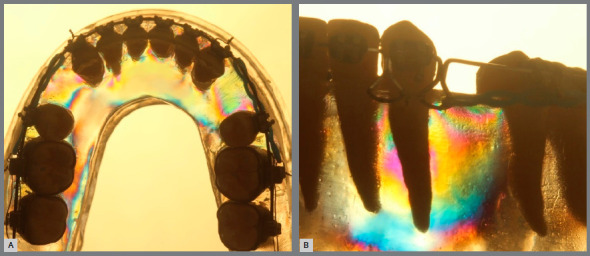
Group 4 photoelastic image: A) occlusal perspective; B) oblique perspective.

» Group 4 (DKL archwire) In the occlusal perspective, moderate intensity color alterations were observed in the canine distal zones. Less intense alterations were observed in the incisor lingual zones when compared to the canine distal zones. In the oblique perspective, mild alterations appeared in the apical zones. On the cervical zones, a more evident increase in fringes was observed in the cervical-distal zone (Fig 9).

**Figure 10:**
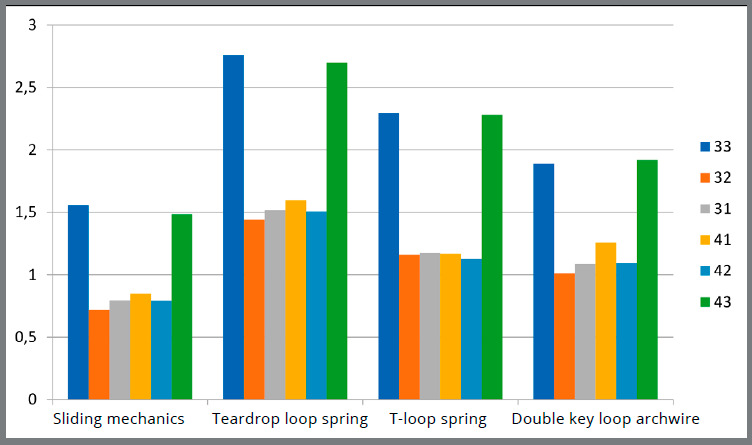
Comparison of the fringe sequence means among the mechanics types in the occlusal perspective.

**Figure 11:**
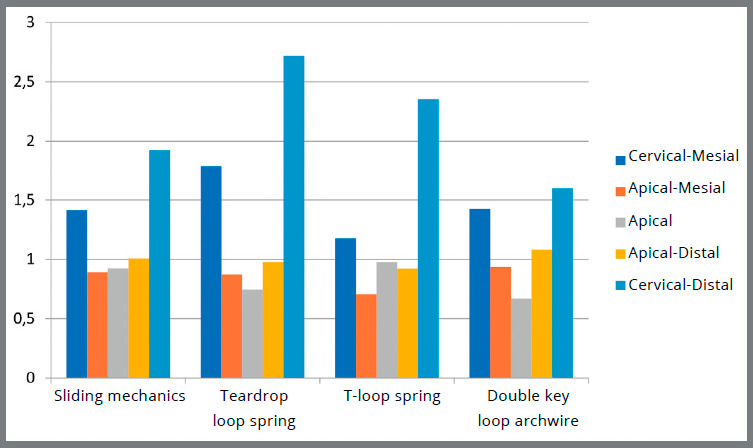
Comparison of the fringe sequence means among the mechanics types in the oblique perspective.

Tables 1 to 4 describe the results obtained from the quantitative analysis. Table 1 describes the mechanics type comparisons according to the previously chosen zones for classification of the fringes in the occlusal perspective. Table 2 describes the comparisons in the oblique perspective. Table 3 shows the paired nonparametric multiple comparisons of the fringes among the mechanics studied in the occlusal perspective. Table 4 shows the comparisons in the oblique perspective. A chart showing the comparison among the fringe sequence means of each mechanical group can be seen in Figure 10 (occlusal perspective) and Figure 11 (oblique perspective).

**Table 1:** Fringe description for each zone of the mechanics types in terms of agreement/repeatability method and the interrater result in the occlusal perspective.

Zone	Mechanics type	Appraisal 1		Appraisal 2		ICC	95% CI	
		Mean	SD	Mean	SD		Lower bound	Upper bound
33	Sliding mechanics	1.5560	0.22909	1.5433	0.21908	0.976	0.932	0.992
	Teardrop loop spring	2.7587	0.21646	2.8187	0.22399	0.753	0.420	0.909
	T-loop spring	2.2967	0.47683	2.4413	0.74272	0.829	0.568	0.939
	Double key loop archwire	1.8887	0.39686	1.9333	0.35670	0.855	0.631	0.948
32	Sliding mechanics	0.7213	0.10623	0.7320	0.13067	0.590	0.115	0.842
	Teardrop loop spring	1.4407	0.25858	1.5087	0.16305	0.801	0.517	0.928
	T-loop spring	1.1593	0.17730	1.2760	0.21836	0.820	0.519	0.937
	Double key loop archwire	1.0100	0.15029	1.0680	0.13832	0.570	0.132	0.828
31	Sliding mechanics	0.7960	0.11350	0.8320	0.10108	0.729	0.364	0.900
	Teardrop loop spring	1.5173	0.16364	1.4607	0.15813	0.871	0.664	0.954
	T-loop spring	1.1733	0.33404	1.2140	0.35020	0.860	0.641	0.950
	Double key loop archwire	1.0873	0.17730	1.0693	0.19326	0.817	0.541	0.934
41	Sliding mechanics	0.8487	0.05680	0.8760	0.10487	0.873	0.671	0.955
	Teardrop loop spring	1.5980	0.16747	1.6107	0.17621	0.959	0.887	0.986
	T-loop spring	1.1680	0.22511	1.2560	0.26362	0.784	0.415	0.925
	Double key loop archwire	1.2560	0.13054	1.3000	0.13732	0.683	0.292	0.880
42	Sliding mechanics	0.7933	0.16101	0.8553	0.14885	0.803	0.363	0.937
	Teardrop loop spring	1.5087	0.16305	1.5467	0.20454	0.784	0.483	0.921
	T-loop spring	1.1280	0.26466	1.1440	0.28727	0.666	0.241	0.875
	Double key loop archwire	1.0947	0.22338	1.0487	0.21139	0.699	0.324	0.887
43	Sliding mechanics	1.4860	0.19526	1.5773	0.37670	0.910	0.756	0.969
	Teardrop loop spring	2.6987	0.34465	2.9607	0.66831	0.672	0.284	0.875
	T-loop spring	2.2787	0.60981	2.3820	0.68833	0.737	0.386	0.903
	Double key loop archwire	1.9200	0.36162	1.7200	0.36162	0.971	0.915	0.990

SD = Standard Deviation; CI= Confidence Interval; ICC = Intraclass Correlation Coefficient.

**Table 2:** Fringe description for each zone of the mechanics types in terms of agreement/repeatability method and the interrater result in the oblique perspective.

Zone	Mechanics type	Appraisal 1		Appraisal 2		ICC	95% CI	
		Mean	SD	Mean	SD		Lower bound	Upper bound
Cervical-mesial	Sliding mechanics	1.4133	0.18711	1.4613	0.20361	0.853	0.604	0.949
	Teardrop loop spring	1.7900	0.33939	1.6827	0.23132	0.735	0.354	0.904
	T-loop spring	1.1773	0.33678	1.2187	0.35236	0.938	0.829	0.979
	Double key loop archwire	1.4273	0.22053	1.3480	0.25585	0.729	0.365	0.900
Apical-mesial	Sliding mechanics	0.8913	0.11186	0.8627	0.11392	0.825	0.552	0.938
	Teardrop loop spring	0.8740	0.16387	0.8847	0.22087	0.688	0.281	0.884
	T-loop spring	0.7067	0.13563	0.8660	0.26981	0.631	0.202	0.858
	Double key loop archwire	0.9353	0.12415	0.9433	0.13026	0.985	0.957	0.995
Apical	Sliding mechanics	0.9233	0.14888	0.9460	0.20152	0.754	0.403	0.910
	Teardrop loop spring	0.7453	0.17225	0.7387	0.16340	0.988	0.966	0.996
	T-loop spring	0.9733	0.29166	1.1087	0.26052	0.551	0.099	0.820
	Double key loop archwire	0.6693	0.28212	0.6733	0.28752	0.686	0.274	0.883
Apical-distal	Sliding mechanics	1.0080	0.09244	1.0040	0.09132	0.986	0.959	0.995
	Teardrop loop spring	0.9740	0.17451	1.0273	0.22369	0.709	0.345	0.891
	T-loop spring	0.9207	0.21569	0.9580	0.20623	0.507	0.010	0.805
	Double key loop archwire	1.0813	0.11325	1.0513	0.13548	0.845	0.595	0.946
Cervical-distal	Sliding mechanics	1.9240	0.13999	2.0160	0.23679	0.535	0.085	0.811
	Teardrop loop spring	2.7220	0.27251	3.1667	0.55635	0.660	0.221	0.873
	T-loop spring	2.3540	0.16530	2.3533	0.22993	0.697	0.293	0.888
	Double key loop archwire	1.6000	0.22370	1.5400	0.25386	0.883	0.645	0.961

SD = Standard Deviation; CI = Confidence Interval; ICC = Intraclass Correlation Coefficient.

**Table 3:** Fringe description of the first evaluation according to mechanics types and comparisons among the groups in the occlusal perspective.

Zone	Mechanics type	Mean	SD	Median	Minimum	Maximum	P
33	Sliding mechanics	1.5560	0.2291	1.3800	1.20	1.81	<0.001
	Teardrop loop spring	2.8587	0.2165	3.0000	2.50	3.10	
	T-loop spring	2.2967	0.4768	2.3300	1.81	3.10	
	Double key loop archwire	1.9887	0.3969	2.0000	1.06	2.50	
32	Sliding mechanics	0.7213	0.1062	0.79	0.60	0.90	<0.001
	Teardrop loop spring	1.4407	0.2586	1.38	0.90	1.81	
	T-loop spring	1.1593	0.1773	1.20	0.79	1.38	
	Double key loop archwire	1.0100	0.1503	0.90	0.79	1.20	
31	Sliding mechanics	0.7960	0.1135	0.79	0.60	0.90	<0.001
	Teardrop loop spring	1.5173	0.1636	1.38	1.38	1.81	
	T-loop spring	1.1733	0.3340	1.38	0.60	1.81	
	Double key loop archwire	1.0873	0.1773	1.06	0.79	1.62	
41	Sliding mechanics	0.8487	0.0568	0.90	0.79	0.90	<0.001
	Teardrop loop spring	1.5980	0.1675	1.62	1.20	1.81	
	T-loop spring	1.1680	0.2251	1.20	0.90	1.62	
	Double key loop archwire	1.2560	0.1305	1.20	1.06	1.38	
42	Sliding mechanics	0.7933	0.1610	0.79	0.60	1.06	<0.001
	Teardrop loop spring	1.5087	0.1630	1.62	1.20	1.81	
	T-loop spring	1.1280	0.2647	0.90	0.90	1.62	
	Double key loop archwire	1.0947	0.2234	1.06	0.79	1.38	
43	Sliding mechanics	1.4860	0.1953	1.38	1.20	1.81	<0.001
	Teardrop loop spring	2.6987	0.3447	2.67	1.81	3.00	
	T-loop spring	2.2787	0.6098	2.33	1.38	3.10	
	Double key loop archwire	1.7200	0.3616	1.62	1.38	2.33	

Friedman test results. SD = Standard Deviation.

**Table 4:** Fringe description of the first evaluation according to mechanics types and comparisons among the groups in the oblique perspective.

Zone	Mechanics type	Mean	SD	Median	Minimum	Maximum	P
Cervical-mesial	Sliding mechanics	1.4133	0.1871	1.38	1.20	2.00	0.0014
	Teardrop loop spring	1.79	0.3394	1.62	1.38	2.33	
	T-loop spring	1.1773	0.3368	1.38	0.60	1.62	
	Double key loop archwire	1.4273	0.2205	1.38	1.00	1.81	
Apical-mesial	Sliding mechanics	0.8913	0.1119	0.90	0.60	1.00	0.0011
	Teardrop loop spring	0.8740	0.1639	0.90	0.60	1.20	
	T-loop spring	0.7067	0.1356	0.60	0.60	1.06	
	Double key loop archwire	0.9353	0.1241	0.90	0.60	1.06	
Apical	Sliding mechanics	0.9233	0.1489	1.00	0.60	1.06	0.0015
	Teardrop loop spring	0.7453	0.1722	0.60	0.60	1.00	
	T-loop spring	0.9733	0.2917	1.06	0.60	1.38	
	Double key loop archwire	0.6693	0.2821	0.45	0.45	1.06	
Apical-distal	Sliding mechanics	1.0080	0.0924	1.06	0.79	1.06	0.0850
	Teardrop loop spring	0.9740	0.1745	0.90	0.79	1.38	
	T-loop spring	0.9207	0.2157	0.90	0.60	1.38	
	Double key loop archwire	1.0813	0.1133	1.06	1.00	1.38	
Cervical-distal	Sliding mechanics	1.9240	0.1400	2.00	1.62	2.00	<0.001
	Teardrop loop spring	2.7220	0.2725	2.50	2.33	3.00	
	T-loop spring	2.3540	0.1653	2.33	2.00	2.50	
	Double key loop archwire	1.6000	0.2237	1.62	1.38	2.00	

Friedman test results. SD = Standard Deviation.

## DISCUSSION

The analysis of the four retraction techniques evaluated in this study revealed interesting results for understanding the behavior of the strains generated during the correction of malocclusions where orthodontic retraction of anterior teeth is indicated. This procedure is greatly studied in the orthodontic literature,^1^ ^-^ ^18^ ^,^ ^22^ ^,^ ^23^ however none of the previous investigations made a linear comparison among the application of these four mechanics.

A body movement of the teeth during anterior retraction allows a more diffuse distribution of the tension in the periodontal ligament and other alveolar tissues, minimizing the risks for cell death and hyalinization of the extracellular matrix, preventing adverse effects, such as root resorption.^30^ When intending to create a final force vector for tooth body movement, the retraction force applied to the anteroinferior teeth was complemented by an additional vertical control, to generate an opposite intrusion moment of force, which attempted to annul the extrusion moment of force caused by the resistance to the retraction in relation to the tooth resistance center, suggesting a final resultant force without intrusion or extrusion of the anterior segment (Fig 5).^4^ ^,^ ^5^ ^,^ ^10^ ^-^ ^15^ For this reason, in the sliding mechanics long hooks were added to the archwire to activate the system close to the tooth resistance center^6^ ^-^ ^8^. For the mechanics applying loops (teardrop loop spring and T-loop spring), a Gable bend was incorporated for each respective loop.^5^ ^-^ ^15^ For the mechanics using the DKL archwire, the mesial loops were tied aiming a deflection of the anterior segment.^16^ ^-^ ^18^

For effective retraction, a correct load of force applied to the anterior teeth is essential. In the literature, varied intensities are found for achieving body tooth movement: 200 g on each side;^5^ 70 g per anterior tooth, thus 210 g of activation for each side;^4^ 240 g on each side for anteroinferior retraction;^10^ ^,^ ^11^ and 150 g to 300 g for a space closure of 0.5 to 1 mm per month.^7^ ^,^ ^8^ This great variation is due to the variety of techniques proposed for such movement. In this study, 240 g of retraction force in each side was applied.

Considering the occlusal perspective, the canine distal zones presented higher strains when compared to the incisors in all evaluated mechanics. This evidence is expected because these teeth are the closest to the point of retraction force, which would lead to a higher incidence of stress on them. When performing the quantitative analysis by examining the means of the fringe sequences, the teardrop loop spring mechanics generated statistically significant higher strains, followed by the T-loop spring, DKL archwire and sliding mechanics. In this perspective, all tensions appearing along the dental root were observed at the same point, because the root was examined longitudinally. Similar studies assessing this perspective could not be found, thus these findings were not discussed. In the oblique perspective, larger strains appeared in the canine cervical-distal (CD) zone in the four evaluated mechanics. Previous photoelastic studies evaluating the anterior segment retraction^17^ and the canine retraction^26^ also observed similar results. Again, the greatest means were observed in the cervical zones when the teardrop loop spring was used. In the apical areas, the differences were small among the groups, and there was no statistically significant difference in the apical-distal (AD) zone. Reviewing the results of this investigation, the safest retraction technique seems to be the sliding mechanics, when compared to the other three evaluated groups. 

The retraction mechanics performed with loops presents a total force obtained from the combination of the retraction force with the intrusion force generated by the Gable bend effect.^4^ ^,^ ^5^ ^,^ ^12^ In the mechanics performed with sliding movement and long hooks, the retraction force is decomposed into two vectors: one related to the retraction force itself and one related to the anterior segment intrusion.^7^ ^,^ ^8^ Therefore, if the same amount of retraction force is applied to the systems, in the mechanic that uses a Gable bend a greater load acts on the teeth, because an additional intrusive force is made. The teardrop loop spring mechanics showing statistically higher strains does not confirm that it is more harmful, but suggests that the orthodontist should be careful when choosing this mechanic, to avoid adverse effects. Further investigations should be performed concerning the differences in the amount of force expressed to the teeth among these retraction mechanics systems with same activation force.

In this study, the strains dissipated in the posterior segments were not evaluated because the contralateral posterior segment of the arc-shaped photoelastic model interposed between the light source and the area supposed to be observed, blocking the correct formation of photoelastic fringes. Several orthodontic anchorage techniques are available according to the required anchorage,^1^ ^,^ ^2^ ^,^ ^4^ ^-^ ^8^ ^,^ ^10^ ^-^ ^18^ ^,^ ^22^ ^,^ ^27^ regardless of using sliding mechanics or loops. Absolute anchorage is not necessarily recommended for all retraction techniques; however, absolute anchorage intends to standardize and reduce the stresses on posterior segment and concentrate these stresses on the anterior teeth roots.^27^ Future studies can be considered to assess the differences in stress dissipation in the anterior and posterior teeth when using different retraction techniques without absolute anchorage.

Photoelasticity is an illustrative approach for assessing the strains produced after the test specimen activation. It was the chosen method for this investigation because the methodology is reproducible and easy to execute,^19^ ^,^ ^25^ which allowed the construction of a study model similar to the evaluated case. It is also widely supported by published studies as an accepted and traditional method to study the tensions acting on the structures of interest.^17^ ^,^ ^19^ ^-^ ^26^ The presence of strains could be assessed and photographed by a direct view of internal structures of the study model. It is considered a periodontal simulator due to the positive correlation to histologic studies.^20^ ^,^ ^21^ Despite several photoelastic studies used only qualitative analysis,^17^ ^,^ ^20^ ^-^ ^24^ this investigation also used a quantitative analysis in which the arrangement of the fringes was visually classified by a sequence of colors, according to the numerical approximation of the values described by the ASTM D4093^25^ ^,^ ^26^, to compare the strain distribution and intensity.

This study allowed the visualization of the strains originated from the activation of retraction movement in several mechanics, providing data to support treatment planning with lower risk of side effects. Finally, it is assumed that *in vitro* method simulating complex structures, like birefringent or numerical models, presents limitations, indicating that the obtained data should be carefully analyzed. More clinical comparisons should be performed to enhance the information available on this topic. 

## CONCLUSION

Reviewing the photoelastic analysis and data for the anterior teeth retraction, greater strains were produced in the distal canine areas (especially cervical). The mean strain of the four mechanics evaluated in ascending order was: sliding mechanics, double key archwire, T-loop spring and teardrop loop spring. The authors conclude that the teardrop loop spring retraction mechanics had the greatest mean strain, and the sliding mechanics had the lowest mean strain on the anterior teeth roots, from both occlusal and oblique perspectives.
